# Draft genome assembly of the slender walking catfish, *Prophagorus nieuhofii*

**DOI:** 10.12688/f1000research.166849.1

**Published:** 2025-08-14

**Authors:** Imron Imron, Fajar Anggraeni, Rahmat Hidayat, Jadmiko Darmawan, Otong Zenal Arifin, Daniel Frikli Mokodongan, Rosita Rosita, Luthfi Nurhidayat

**Affiliations:** 1 Research Center for Fishery, National Research and Innovation Agency Republic of Indonesia, Cibinong, West Java, 16915, Indonesia; 2 Research Center for Applied Zoology, National Research and Innovation Agency Republic of Indonesia, Cibinong, West Java, 16915, Indonesia; 3 Research Center for Biosystematics and Evolution, National Research and Innovation Agency Republic of Indonesia, Cibinong, West Java, 16915, Indonesia; 4 Department of Fisheries, Faculty of Agriculture, Palangka Raya University, Palangka Raya, Central Kalimantan, Indonesia; 5 Faculty of Biology, Universitas Gadjah Mada, Sleman, DI Yogyakarta, 55281, Indonesia

**Keywords:** Whole genome sequencing, genome assembly, genome annotation, slender walking catfish, Prophagorus nieuhofii.

## Abstract

The slender walking catfish, *Prophagorus nieuhofii*, plays an important role in small-scale fisheries across Southeast Asia, supporting food security. While IUCN currently lists it as a Least Concern species, growing demand and pressures such as overfishing, habitat loss, and degradation may elevate its conservation risk. To support sustainable fisheries management and aquaculture, we sequenced, assembled, and annotated the whole genome of this species. The specimen was part of an expedition to document and preserve the genetic resources of aquatic animals in Kalimantan’s freshwater ecosystems. Using 27 Gb of sequence data, we assembled a 1.1 Gb genome comprising 5,790 scaffolds. This genome assembly has high contiguity and completeness, with N50 of 33.7 Mb and a BUSCO score of 98.8%. Repeat annotation revealed that 48.17% of the genome consisted of repetitive elements, predominantly DNA transposons (18.56%) and retroelements (13.30%). Structural annotation identified 30,099 protein-coding genes and 37,734 transcripts, most of which were multi-exonic and rich in alternative splicing. BUSCO analysis confirmed the high completeness of the genome and annotation, with 97.7% of the conserved orthologs being detected.

## Introduction

The slender walking catfish, *Prophagorus nieuhofii* (previously known as *Clarias nieuhofii*), is widespread in Southeast Asia, including Indonesia –specifically Java, Sumatra, and Kalimantan–, the Malay Peninsula, Singapore, Thailand, and the Philippines. ^1^ It is a popular food fish due to its good taste and nutritional benefits and is an important species for food security by supporting artisanal fisheries. While the IUCN Red List of threatened species classifies it as Least Concern in the global assessment of species conservation, habitat loss and degradation and fishing pressure have resulted in a decline in many natural populations. ^2– 4^ In Thailand, it has been classified as a vulnerable species, ^5^ and a genetic assessment has been carried out to manage its natural populations. ^4^

In addition to maintaining the sustainability of this fish population in its natural environment, several studies have been conducted to develop it into a farmed species. This species, owing to its air-breathing capability, resilience, and adaptability, shows significant potential for domestication and aquaculture. Preliminary studies on domestication and aquaculture have been conducted. These included the study of growth and survival during the early stages of domestication, ^6^ breeding and reproduction ^7^ and exploration as a probiotic source. ^8^ Although further research is necessary to optimize its cultivation, its inherent characteristics are conducive to successful aquaculture.

Generation of the whole genome sequence of this species will provide a good resource for both fisheries management and aquaculture development. In the former case, the large discovery of single nucleotide polymorphisms (SNPs) that cover genome wide (neutral) and allele-specific (adaptive) diversity patterns will provide a good resource for genomic stock identification, traceability, fisheries-induced evolution and climate change. ^9^ In the latter case, the whole genome sequence, combined with other technologies, such as quantitative trait loci (QTL) analysis, genome-wide association studies (GWAS), and expression profiling, allowing for the prediction of genotypic variants associated with phenotypic traits, can be used to improve traits in breeding programs. ^10, 11^

## Methods

### Sample collection, DNA extraction, and genome sequencing

Fish samples were collected during a 2024 expedition aimed at characterizing genetic resources of aquatic animals from a natural population in South Kalimantan, Indonesia (3°21′43.0″S, 114°42′08.3″E). Specimen were captured using bubu traps and held in a pond with 60 cm water depth at 27-28°C for three days to reduce stress. Prior to DNA tissue sampling, fish were anesthetized following ^12^: they were placed in a 35-liter bucket with 7 cm of water at 28°C, cooled with liquid ice to 21°C, and then clove oil was added at 160 mg/L. Tissue samples were collected when the fish showed minimal movement after anesthesia. A 10 mg tissue sample was collected from an individual measuring of 32.5 cm in length and weighing 277 g, preserved in DNA shield solution and transported to the laboratory for sequencing. High-quality DNA was extracted using the Quick-DNA high molecular weight (HMW) MagBead kit (Zymo Research) with overnight proteinase K digestion incubation. The DNA extract was quantified using a Qubit fluorometer with an Equalbit 1x ds-DNA HS assay kit for sequencing.

Whole genome sequencing was performed using Oxford Nanopore Technology (ONT) – PromethION. Genomic DNA (1500 ng DNA in 48uL nuclease free water was incubated at 20°C for 30 min, followed by incubation at 65°C for 5 min. Sequencing by ligation was performed using the Ligation Sequencing DNA V14 workflow kit (SQK-LSK114). The basecaller tool was Dorado v0.9.1, using dna_r10.4.1_e8.2_400bps_sup@v5.0.0 basecalling model, with a minimum Q score of 10 and trimming of adapters and barcodes. The quality of the sequencing data was checked using NanoPlot. ^13^

### Genome assembly and annotation

Genome assembly estimation was done using Flye 2.9.5, ^14^ while genome scaffolding was conducted with RagTag 2.1.0 ^15^ guided by the reference genome of *Clarias gariepinus* (GCF_024256425.1). Genome size was estimated using Jellyfish software version 2.3.1 ^16^ and further processed with GenomeScope 2.0 v2.0.1. The assembly statistics were calculated using assembly-stat version 1.0.1. The completeness of the assembly was estimated using Benchmarking Universal Single-Copy Orthologous (BUSCO) version 5.8.2, utilizing miniport. ^17– 19^

Repetitive elements within the genome assembly were identified using RepeatModeler v2.0.6 in conjunction with RepeatMasker v4.1.7 ( http://www.repeatmasker.org). Prior to annotation, these repetitive regions were soft masked to minimize interference. Structural genome annotation encompassing gene prediction was conducted using the GALBA pipeline, ^20^ which employs miniprot ^17^ and AUGUSTUS, ^21^ integrating protein data from closely related species as extrinsic evidence. Specifically, protein data from *Clarias gariepinus* (GCF_024256425.1), *Ictalurus furcatus* (GCF_023375685.1), *Ictalurus punctatus* (GCF_001660625.3), and *Tachysurus fulvidraco* (GCF_022655615.1) were utilized. Functional annotation of the resulting gene predictions was then performed using the ‘funannotate annotate’ command from the Funannotate pipeline ( https://funannotate.readthedocs.io/en/latest/install.html), incorporating tools such as InterProScan5, ^22^ Eggnog-Mapper, ^23^ and SignalP 5.0 ^24^ to assign gene names and predict protein functions. Finally, the completeness of the genome annotation was evaluated using BUSCO v5.8.2. ^19^

### Ethical approval

This research was approved by the Ethics Commission for Animal Husbandry and Use, National Research and Innovation Agency (Approval No. 174/KE.02/SK/07/2024). All animal-related procedures were conducted in accordance with institutional guidelines and complied with the ARRIVE 2.0 reporting standards, the checklists for which are available at https://doi.org/10.6084/m9.figshare.29612615.v1. ^25^

## Results

### Sequence output and genome assembly

**Figure 1.**
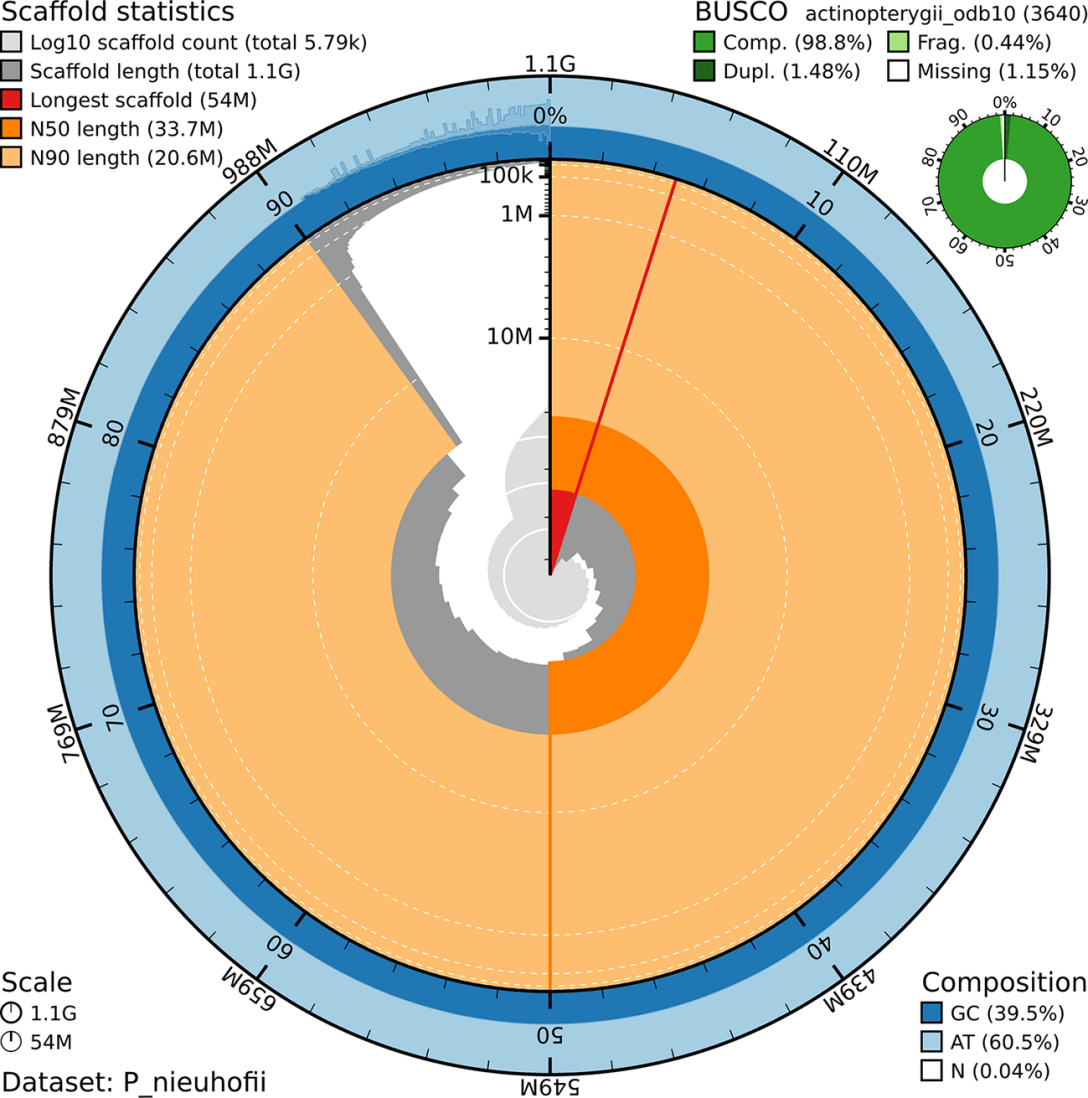
A snail graph showing the main features of *Prophagorus nieuhofii* genome assembly.

Sequencing produced a total of 27,388,841,658 bases from 4,440,560 reads, with 99.8% of bases meeting the designated quality standards. The highest observed mean basecall quality score was 46.4 with a read length of 133, while the longest read reached 6,129,425 with a mean basecall quality score of 14.5 ( Table 1). The draft of genome assembly, as illustrated in the snail graph ( Figure 1), comprises approximately 5,790 scaffolds, totaling 1.1 gigabases, with the longest scaffold of 54 spanning megabases.

**Table 1.** Summary statistics of sequences of slender walking catfish ( *Prophagorus nieuhofii*).

Total bases (bases)	27,388,841,658.0
Mean read length	6,167.9
Mean read quality	20.2
Median read length	4,772.0
Median read quality	23.6
Number of reads	4,440,560
Read length (N50)	8,687.0
STDEV read length	5,796.8

The N50 and N90 values, measuring assembly continuity, are 33.7Mb and 20.6Mb, respectively. The base composition showed 39.5% GC content and 60.5% AT content, whereas the N content (gaps) remained minimal at 0.04%, indicating a highly contiguous and well-assembled genome. Using the Actinopterygii ortholog database, which is based on 3640 universal genes, the assembly demonstrated 98.8% completeness with a low percentage of missing BUSCO (1.15%), suggesting that most expected genes are present. The genome size of this species is similar to that of a related species, *Clarias gariepinus*, which has a genome size of 969.62 Mb and contig N50 of 33.71 Mb. ^26^ Genome composition based on a 21-mer based characterization shows a heterozygosity rate of 0.78%, while the homozygosity rate was 99.12%.

### Genome annotation

**Repeats annotation**

Repeat annotation analysis revealed that approximately 48.17% of the genome (529,073,132 bp) consisted of repetitive elements ( Table 2). Among these, retroelements accounted for 13.30% of the genome, spanning over 146 million base pairs across 492,154 elements. This category includes SINEs, which comprise 1.93% of the genome, and LINEs, the largest subgroup of retroelements, which occupy 5.33%. The LINEs were mainly composed of L2/CR1/Rex elements (4.01%), followed by the R1/LOA/Jockey, RTE/Bov-B, and L1/CIN4 subfamilies. LTR elements were also prominent, comprising 6.04% of the genome, largely represented by Gypsy/DIRS1 (2.55%) and retroviral elements (1.05%), along with smaller contributions from BEL/Pao and Ty1/Copia. Notably, some retroelement families such as CRE/SLACS were not detected.

**Table 2.** Classification of repeat elements of the slender walking catfish ( *Prophagorus nieuhofii*) genome assembly.

Repeat category	Count	Occupied bp	% of Genome
Retroelements	492,154	146,089,956	13.30%
SINEs	104,157	21,222,223	1.93%
Penelope	3,786	390,741	0.04%
LINEs	170,940	58,512,142	5.33%
CRE/SLACS	0	0	0.00%
L2/CR1/Rex	115,148	43,988,622	4.01%
R1/LOA/Jockey	15,957	3,985,162	0.36%
R2/R4/NeSL	343	218,349	0.02%
RTE/Bov-B	11,503	2,944,056	0.27%
L1/CIN4	8,590	3,281,609	0.30%
LTR elements	217,057	66,355,591	6.04%
BEL/Pao	4,144	3,425,287	0.31%
Ty1/Copia	93	112,540	0.01%
Gypsy/DIRS1	51,836	27,984,797	2.55%
Retroviral	43,638	11,543,937	1.05%
DNA Transposons	924,291	203,850,283	18.56%
hobo-Activator	163,993	37,319,984	3.40%
TC1-IS630-Pogo	581,362	124,970,541	11.38%
En-Spm	0	0	0.00%
MULE-MuDR	1,323	99,951	0.01%
PiggyBac	13,967	4,552,618	0.41%
Tourist/Harbinger	25,580	7,005,418	0.64%
Other (e.g. Mirage, P- element, Transib)	4,950	5,389	0.04%
**Rolling-circles**	30,262	6,192,298	0.56%
**Unclassified**	986,211	133,225,464	12.13%
**Total Interspersed**		483,556,444	44.03%
**Small RNA**	57,989	13,482,676	1.23%
**Satellites**	1,781	570,181	0.05%
**Simple repeats**	773,672	34,790,247	3.17%
**Low complexity**	59,081	3,378,970	0.31%
**Total Masked Bases**		529,073,132	48.17%

DNA transposons represented the largest category of repeats, both in number and genomic coverage, with 924,291 elements occupying 18.56% of the genome (203.9 million base pairs). Within this group, the TC1-IS630-Pogo family was predominant, covering 11.38% of the genome. Other notable contributors included hobo-Activator (3.40%), PiggyBac (0.41%), Tourist/Harbinger (0.64%), and MULE-MuDR (0.01%), while some families, such as En-Spm, showed no representation. Additionally, rolling-circle transposons comprising 30,262 elements and 0.56% of the genome were identified. A substantial portion of the genome (12.13%) contained unclassified elements, amounting to 986,211 entries. These may represent novel, divergent, or currently uncategorized repeat families.

Other repetitive elements included small RNA-related sequences (1.23%), simple repeats (e.g. microsatellites, 3.17%), low-complexity regions (0.31%), and satellite DNA (0.05%). Overall, interspersed repeats alone account for 44.03% of the genome (483.6 million base pairs), underscoring the genomic complexity and abundance of repetitive sequences, especially DNA transposons and retroelements.

**Structural and functional annotation**

The genome annotation process resulted in the identification of 30,099 protein-coding genes, which in turn produced 37,734 predicted transcripts with an average of 1.3 transcripts per gene ( Table 3). Alternative splicing was observed in 5,459 of these genes. The majority of genes (87.5%, corresponding to 26,327 genes) were found to be multi-exonic, whereas the remaining 12.5% were composed of a single exon. Each gene spans an average locus length of 15,993.4 base pairs, measured from the first exon to the last exon. On average, genes are composed of 8.9 distinct exons, with a total of 268,395 exons annotated across the genome. The mean exon size was 180.2 bp, and the average transcript size, inclusive of UTRs and coding regions, was 1,812.4 bp.

**Table 3.** Genome annotation summary of the slender walking catfish ( *Prophagorus nieuhofii*) genome assembly.

Annotation features	Values
Max number of transcripts per gene	8
Mean exon size	180.2
Mean gene locus size (first to last exon)	15993.4
Mean number of distinct exons per gene	8.9
Mean number of transcripts per gene	1.3
Mean transcript size (UTR, CDS)	1812.4
Number of distinct exons	268,395
Number of genes	30,099
Number of genes with alternative transcript variants	5,459 (18.1%)
Number of multi-exon genes	26,327 (87.5%)
Number of predicted transcripts	37,734
Number of single-exon genes	3,772 (12.5%)

Regarding genome composition, exons constitute 4% of the genome, spanning approximately 48 Mb, with a GC content of 51% ( Table 4). Genes collectively occupy 44% of the genome, covering approximately 481 Mb, and have a GC content of 40%. The median length of annotated genes was 6,955 bp. Introns accounted for an additional 40% of the genome, totaling 434 Mb. Their average length was 1,861 bp, a median of 481 bp, and a GC content of 39%. In total, 233,110 introns were identified.

**Table 4.** Genome composition of the slender walking catfish ( *Prophagorus nieuhofii*) genome assembly.

	% GC	% of genome	Average size (bp)	Median size (bp)	Number	Total length (Mb)
Exon	51%	4%	180	126	268,395	48 Mb
Gene	40%	44%	15,993	6,955	30,099	481 Mb
Intron	39%	40%	1,861	481	233,110	434 Mb

**Figure 2.**
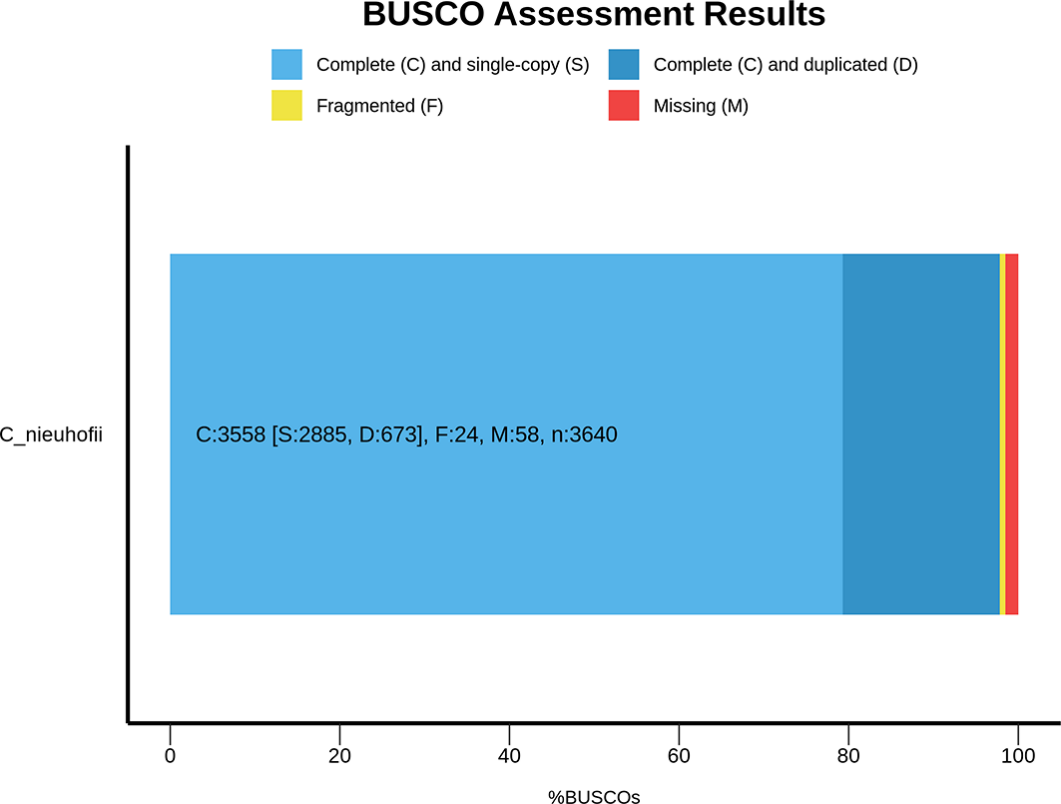
BUSCO assessment of genome annotation results for the slender walking catfish ( *Prophagorus nieuhofii)* genome assembly.

To assess the completeness of the annotation, BUSCO analysis was conducted using the actinopterygii_odb10 lineage dataset. The analysis revealed that 97.7% of the 3,640 expected single-copy orthologs were complete, with 79.3% identified as single-copy and 18.5% as duplicated BUSCOs ( Figure 2). Only 0.7% were fragmented and 1.6% were missing, indicating a highly complete and well-annotated gene set. The high BUSCO score highlights the robustness of the genome annotation, affirming its appropriateness for subsequent biological and comparative analyses.

## Data Availability

The project contains two underlying data:
1. The raw whole genome sequence. 2. The genome assembly. The raw whole genome sequences are available on NCBI’s Short Read Archive (SRA): Whole genome sequence of *Prophagorus nieuhofii*, accession number: SRR34064805 ( https://www.ncbi.nlm.nih.gov/sra/?term=SRR34064805). The raw sequences were also deposited and are accessible on the Zenodo repository: Data set for draft genome assembly of the slender walking catfish, *Prophagorus nieuhofii* DOI: https://doi.org/10.5281/zenodo.16689652. ^27^ The genome assembly data were deposited and made accessible to Dataverse: Replication data for draft genome assembly of the slender walking catfish, *Prophagorus nieuhofii.* https://hdl.handle.net/20.500.12690/RIN/ULQDHU. ^28^ All the underlying data of this study are openly available under the terms of Creative Commons Zero v1.0 (CC0 1.0) Universal Public Domain Dedication.
