# Supplementary material for: Reference-guided draft genome assembly of the slender walking catfish, Prophagorus nieuhofii
Source: F1000Res. 2025 Nov 10;14:787. Originally published 2025 Aug 14. [Version 2] doi: 10.12688/f1000research.166849.2 (PMC12743208; doi:10.12688/f1000research.166849.2)
Supplement: Peer review history

# Reviewer report: Reviewer response for version 2

**DOI:** 10.5256/f1000research.190588.r436285

**Published:** 2025-12-26

**Authors:** Adauto Cardoso

**Affiliations:** 1 Universidade Estadual Paulista, Botucatu, Brazil

This study describes the sequencing, assembly, and annotation of a first version of the genome of the species *Prophagorus nieuhofii*, a species of economic and food health relevance. The methods employed are well described, and the results are clearly presented. Although the work presents some limitations related to coverage and completeness of the assembly, the data presented are relevant and may have diverse biological, evolutionary and biotechnological applications. Efforts to present genomic data for species still unknown in this respect should be considered, although improvements should be sought in the future. My only major concern is the lack of more detailed characterization of satellite DNAs, even though computational tools are available to detect them in long-reads.

Are the datasets clearly presented in a usable and accessible format, and the assembly and annotation available in an appropriate subject-specific repository?

Yes

Are sufficient details of the sequencing and extraction, software used, and materials provided to allow replication by others?

Yes

Are the rationale for sequencing the genome and the species significance clearly described?

Yes

Are the protocols appropriate and is the work technically sound?

Yes

Reviewer Expertise:

Cytogenomics, Genomics

I confirm that I have read this submission and believe that I have an appropriate level of expertise to confirm that it is of an acceptable scientific standard.

# Reviewer report: Reviewer response for version 1

**DOI:** 10.5256/f1000research.183897.r411495

**Published:** 2025-09-27

**Authors:** Haiyang Liu

**Affiliations:** 1 Pearl River Fisheries Research Institute, Guangzhou, China

This manuscript reports the first draft genome assembly and annotation of the slender walking catfish ( *Prophagorus nieuhofii*). Given the ecological and economic importance of this species, generating genomic resources is valuable for fisheries management, germplasm utilization, and molecular breeding. The study provides initial genomic data and methodological details, but there are notable shortcomings in sequencing depth, assembly strategy, biological interpretation, and data usability. These limitations reduce its utility as a long-term genomic reference resource.

 1. The reported dataset (~27 Gb) represents <30× coverage. For reliable long-read assemblies (ONT or PacBio), ≥30×—ideally >50×—coverage is generally recommended. The relatively low coverage increases risks of collapsed repeats, gaps, and structural errors, which compromise annotation accuracy.

 2. Scaffolding was performed using *Clarias gariepinus* as the reference. Reference-guided assembly may introduce biases and misassemblies, especially if structural rearrangements exist between species. Independent scaffolding methods (e.g., Hi-C, optical mapping, or ultra-long ONT reads) are needed to improve reliability. The manuscript title should explicitly indicate that this is a **reference-guided assembly**.

 3. Gene annotation results are presented mainly as averages (gene length, exon number, etc.), which lack biological interpretation. Comparisons with other catfish genomes would strengthen the significance of these findings.

Are the datasets clearly presented in a usable and accessible format, and the assembly and annotation available in an appropriate subject-specific repository?

Yes

Are sufficient details of the sequencing and extraction, software used, and materials provided to allow replication by others?

Yes

Are the rationale for sequencing the genome and the species significance clearly described?

Yes

Are the protocols appropriate and is the work technically sound?

Yes

Reviewer Expertise:

genomics, genetics, breeding,

I confirm that I have read this submission and believe that I have an appropriate level of expertise to confirm that it is of an acceptable scientific standard, however I have significant reservations, as outlined above.

# Reviewer report: Reviewer response for version 1

**DOI:** 10.5256/f1000research.183897.r405799

**Published:** 2025-09-16

**Authors:** Geoffrey C. Waldbieser

**Affiliations:** 1 USDA-ARS Warmwater Aquaculture Research Unit, Stoneville, USA

This Genome Note describes an assembly of Nanopore sequence from the catfish species Prophagorus nieuhofii. The rationale for sequencing the genome and the significance of this species is well presented. The Nanopore sequencing, assembly, and repeat annotation are described sufficiently to be replicated. The fasta sequence of the genome is available but there is no available annotation of the scaffolds.

 The contigs were scaffolded based on the Claris gariepinus reference genome. This does not mean the assembly is correct. The authors point to two other Ictalurus catfish genomes, and the first genome assembly of Ictalurus furcatus was a reference based assembly based on Ictalurus punctatus. However, an independent assembly of I. furcatus revealed three major chromosomal inversions between the two species that did not show up on the reference-guided assembly. The title should reflect this is a reference-based assembly. The assembly will still be useful for some molecular marker approaches - but they should be cautious about QTL and GWAS experiments.

 Page 4: What is the minimum length of sequence put into the assembler? 

 Figures 1 and 2 add no value to the data already presented in the text and tables. Both figures should be removed.

 Average values are used for gene length, etc, but those are just mathematical calculations and may not mean anything biological. How do these numbers compare with the annotation of other catfish genomes?

Are the datasets clearly presented in a usable and accessible format, and the assembly and annotation available in an appropriate subject-specific repository?

Partly

Are sufficient details of the sequencing and extraction, software used, and materials provided to allow replication by others?

Yes

Are the rationale for sequencing the genome and the species significance clearly described?

Yes

Are the protocols appropriate and is the work technically sound?

Partly

Reviewer Expertise:

Molecular biology, genomics, genome assembly

I confirm that I have read this submission and believe that I have an appropriate level of expertise to confirm that it is of an acceptable scientific standard, however I have significant reservations, as outlined above.

# Response

**Published:** 2025-10-30

**Authors:** Imron Imron

**Affiliations:** Research Center for Fishery, National Research and Innovation Agency, Bogor, West Java, Indonesia

- This Genome Note describes an assembly of Nanopore sequence from the catfish species Prophagorus nieuhofii. The rationale for sequencing the genome and the significance of this species is well presented. The Nanopore sequencing, assembly, and repeat annotation are described sufficiently to be replicated. The .fasta sequence of the genome is available but there is no available annotation of the scaffolds. 

**Response: **Thank you for the comments. We are sorry that we missed the annotation file within the manuscript. We have now provided link to the annotation results within the manuscript in Data availability section:   

 “....The genome annotation data were deposited and made accessible in the Zenodo repository: Genome annotation of the previously assembled genome of the slender walking catfish, *Prophagorus nieuhofii* (DOI https://doi.org/10.5281/zenodo.17422526)” 

- The contigs were scaffolded based on the Claris gariepinus reference genome. This does not mean the assembly is correct. The authors point to two other Ictalurus catfish genomes, and the first genome assembly of Ictalurus furcatus was a reference-based assembly based on Ictalurus punctatus. However, an independent assembly of I. furcatus revealed three major chromosomal inversions between the two species that did not show up on the reference-guided assembly. The title should reflect this is a reference-based assembly. The assembly will still be useful for some molecular marker approaches - but they should be cautious about QTL and GWAS experiments. 

**Response: **Thank you for the comments. We recognize the potential for misassembly when using a reference-guided approach. We also agree that the genome assembly will remain valuable for other research involving coding regions. Consequently, we have revised the title of this manuscript, as suggested by the reviewer, to “Reference-guided draft genome assembly of the slender walking catfish, *Prophagorus nieuhofii.*” 

  

- Page 4: What is the minimum length of sequence put into the assembler?  

**Response:**  We used 3286 bp as a minimum length for the sequence put into assembler. This number was determined based on the N90 value of our raw sequences.  

- Figures 1 and 2 add no value to the data already presented in the text and tables. Both figures should be removed. 

**Response:** We have removed the figure 1 and 2 from the main text and moved them into the Zoenodo repository as extended data   

  

- Average values are used for gene length, etc, but those are just mathematical calculations and may not mean anything biological. How do these numbers compare with the annotation of other catfish genomes? 

**Response:**  Thank you for the comments. We have consolidated Table 3 and Table 4 into comparative tables, which now include a comparison with the genome assemblies of four other catfish species: *Clarias gariepinus (Clariidae), Ictalurus furcatus (Ictaluridae), Ictalurus punctatus (Ictaluridae), *and *Tachysurus fulvidraco (Bagridae)*. As a result, we have revised the text in the “Structural and Functional Annotation” subsection to enhance the comparative description. 

 “The genome annotation of *Prophagorus nieuhofii *resulted in the identification of 30,099 protein-coding genes, a count comparable to that of *Ictalurus punctatus* (approx 31,040 genes), which produced 37,734 predicted transcripts. Relative to the other four catfish species examined (Table 3), *P. nieuhofii* exhibits a compact gene architecture and reduced splicing complexity. This is evidenced by the lowest mean number of transcripts per gene at 1.3 (others range from 1.7 to 2.3), and alternative splicing detected in only 18.1% of genes, significantly less than the 32.2% to 48.0% observed in the remaining species. Furthermore, *P. nieuhofii *has a substantially higher proportion of single-exon genes (12.5%), far exceeding the 3.5% to 4.4% found in the other catfish, which possess a more uniformly multi-exonic architecture. Structural measurements confirm this compactness: *P. nieuhofii* genes have a smaller average locus length (15,993.4 bp vs. 19,155.7 bp to 22,083.0 bp) and fewer distinct exons per gene (8.9 vs. 11.3 to 12.3). Its mean exon size is also the smallest at 180.2 bp (others range from 269.4 bp to 327.1 bp), resulting in a significantly shorter average transcript size (1,812.4 bp). 

 In terms of genome composition (Table 4), the *P. nieuhofii* genome has the lowest overall content dedicated to coding and genic regions. Exons constitute only 4% of the genome (48 Mb), which is notably low compared to the 8% to 13% seen elsewhere but exhibit the highest GC content at 51% (compared to 45%─46% in the others). Genes collectively occupy 44% of the genome (481 Mb), marking the smallest genic fraction (others range from 55% to 68%). Introns, totaling 233,110, account for 40% of the genome (434 Mb) with an average length of 1,862 bp, also representing the lowest proportional content in the comparison. The structural and compositional differences collectively indicate that the *P. nieuhofii *genome exhibits a more compact gene structure and less complexity in transcript diversity compared to the other catfish assemblies studied.”

# Reviewer report: Reviewer response for version 1

**DOI:** 10.5256/f1000research.183897.r413914

**Published:** 2025-09-16

**Authors:** Gulab D. Khedkar

**Affiliations:** 1 Dr. Babasaheb Ambedkar Marathwada University, Aurangabad, Maharashtra, India

**Review report**

*Overall Assessment*

 The manuscript presents the first draft genome assembly of *Prophagorus nieuhofii* (slender walking catfish), an ecologically and economically important species in Southeast Asia. The study addresses an ecologically and economically relevant species, and I appreciate the effort to make genomic resources available for a taxon of regional importance. However, after a careful evaluation, I have major concerns that prevent me from recommending acceptance in its current form.

 Detailed Assessment

*a. Sequencing depth appears insufficient*

 The reported coverage is below the accepted standards for reliable genome assembly (generally ≥30× long-read depth). Low coverage increases the likelihood of collapsed repeats, gaps, and structural errors, which in turn reduce the reliability of annotations and downstream analyses.

*b. Assembly completeness is limited*

 The assembly is fragmented and scaffolded with reliance on a reference genome ( *Clarias gariepinus*). This strategy risks introducing misassemblies and biases. Independent scaffolding methods such as Hi-C, optical mapping, or ultra-long ONT reads are required to approach chromosome-level quality, which is expected for a lasting genomic reference. Although the BUSCO score is high, it does not fully compensate for the lack of structural completeness.

*c. Limited utility of the current resource*

 A genome note should provide a robust foundation for comparative genomics, conservation, and applied research. In its present form, the assembly’s limitations significantly restrict its long-term usefulness. The manuscript would be strengthened by deeper sequencing, improved scaffolding, and expanded biological interpretation (e.g., comparative analysis with related catfish species).

*d. Minor points*

 Please consider providing a clear summary table that links raw sequencing datasets to analytical steps (assembly, polishing, annotation). Functional annotation could be expanded to highlight gene families of ecological or aquacultural interest.

*e. Decision*

 While the study has potential, I regret to say that the present version does not meet the standards required for indexing as a genome note. I therefore recommend rejection in its current form. I encourage the authors to consider resequencing at higher depth and applying independent scaffolding approaches. With these improvements, the dataset could provide a far more valuable and reliable genomic resource.

Are the datasets clearly presented in a usable and accessible format, and the assembly and annotation available in an appropriate subject-specific repository?

No

Are sufficient details of the sequencing and extraction, software used, and materials provided to allow replication by others?

Partly

Are the rationale for sequencing the genome and the species significance clearly described?

Yes

Are the protocols appropriate and is the work technically sound?

Partly

Reviewer Expertise:

Evolutionary biology, genomics, population genetics

I confirm that I have read this submission and believe that I have an appropriate level of expertise to state that I do not consider it to be of an acceptable scientific standard, for reasons outlined above.

# Response

**Published:** 2025-10-30

**Authors:** Imron Imron

**Affiliations:** Research Center for Fishery, National Research and Innovation Agency, Bogor, West Java, Indonesia

Detailed Assessment 

 a. Sequencing depth appears insufficient 

 The reported coverage is below the accepted standards for reliable genome assembly (generally ≥30× long-read depth). Low coverage increases the likelihood of collapsed repeats, gaps, and structural errors, which in turn reduce the reliability of annotations and downstream analyses. 

**Response: **Thank you for your comments. We fully acknowledge the necessity of higher coverage for creating a reliable genome assembly. However, due to certain limitations, including funding constraints, we were unable to achieve more than 30x coverage. Nonetheless, if given the opportunity in the future, we would like to enhance the genome assembly. 

 b. Assembly completeness is limited 

 The assembly is fragmented and scaffolded with reliance on a reference genome ( *Clarias gariepinus*). This strategy risks introducing misassemblies and biases. Independent scaffolding methods such as Hi-C, optical mapping, or ultra-long ONT reads are required to approach chromosome-level quality, which is expected for a lasting genomic reference. Although the BUSCO score is high, it does not fully compensate for the lack of structural completeness. 

**Response:** We thank the reviewer for this valuable comment and fully agree that independent scaffolding methods such as Hi-C, optical mapping, or ultra-long ONT reads would improve the structural completeness of the assembly. The primary goal of this work, however, was to generate a high-quality draft genome for *Prophagorus nieuhofii* as a foundational resource, rather than a chromosome-level reference assembly. 

 We have revised the manuscript title to “Reference-guided draft genome assembly of the slender walking catfish, *Prophagorus nieuhofii*” in order to be transparent about using a reference-guided approach based on the closely related *Clarias gariepinus* genome. This strategy was chosen because Hi-C and ultra-long read sequencing technologies are currently limited in availability within our country, and reference-based scaffolding remains a practical and widely accepted alternative for non-model species under such constraints. While we acknowledge that reference-guided scaffolding can introduce some degree of structural bias, the assembly’s high contiguity (N50 = 33.7 Mb), completeness (BUSCO = 98.8%), and minimal gap content (0.04%) demonstrate its robustness and reliability for downstream genomic and comparative analyses.  

 c. Limited utility of the current resource 

 A genome note should provide a robust foundation for comparative genomics, conservation, and applied research. In its present form, the assembly’s limitations significantly restrict its long-term usefulness. The manuscript would be strengthened by deeper sequencing, improved scaffolding, and expanded biological interpretation (e.g., comparative analysis with related catfish species). 

**Response: **We thank the reviewer for this thoughtful comment and agree that deeper sequencing, improved scaffolding, and comparative analyses with related catfish species would further strengthen this genomic resource. Our study focused on generating a high-quality draft genome as an initial reference for *Prophagorus nieuhofii*. We acknowledge that additional long-range scaffolding and deeper sequencing would enhance structural resolution, and we plan to pursue these improvements as such technologies become more accessible in our country. 

 While we appreciate the suggestion for broader comparative analyses, the genome note format emphasizes concise presentation of sequencing and assembly results. Nonetheless, we have included annotation statistics and genome composition comparisons with four related catfish species *(Clarias gariepinus, Ictalurus furcatus, Ictalurus punctatus, and Tachysurus fulvidraco)*, consistent with the scope of this article type.

 1. The reported dataset (~27 Gb) represents <30× coverage. For reliable long-read assemblies (ONT or PacBio), ≥30×—ideally >50×—coverage is generally recommended. The relatively low coverage increases risks of collapsed repeats, gaps, and structural errors, which compromise annotation accuracy. 

 Response: Thank you for your comments. We fully acknowledge the necessity of higher coverage for creating a reliable genome assembly. However, due to certain limitations, including funding constraints, we were unable to achieve more than 30x coverage. Nonetheless, if given the opportunity in the future, we would like to enhance the genome assembly. 

 2. Scaffolding was performed using Clarias gariepinus as the reference. Reference-guided assembly may introduce biases and misassemblies, especially if structural rearrangements exist between species. Independent scaffolding methods (e.g., Hi-C, optical mapping, or ultra-long ONT reads) are needed to improve reliability. The manuscript title should explicitly indicate that this is a reference-guided assembly. 

 Response: Thank you for the comments. We recognize the potential for misassembly when using a reference-guided approach. Consequently, we have revised the title of this manuscript, as suggested by the reviewer, to “Reference-guided draft genome assembly of the slender walking catfish, Prophagorus nieuhofii.” 

 3. Gene annotation results are presented mainly as averages (gene length, exon number, etc.), which lack biological interpretation. Comparisons with other catfish genomes would strengthen the significance of these findings. 

 Response:  Thank you for the comments. We have consolidated Table 3 and Table 4 into comparative tables, which now include a comparison with the genome assemblies of four other catfish species: Clarias gariepinus (Clariidae), Ictalurus furcatus (Ictaluridae), Ictalurus punctatus (Ictaluridae), and Tachysurus fulvidraco (Bagridae). As a result, we have revised the text in the “Structural and Functional Annotation” subsection to enhance the comparative description. 

 “The genome annotation of Prophagorus nieuhofii resulted in the identification of 30,099 protein-coding genes, a count comparable to that of Ictalurus punctatus (approx 31,040 genes), which produced 37,734 predicted transcripts. Relative to the other four catfish species examined (Table 3), P. nieuhofii exhibits a compact gene architecture and reduced splicing complexity. This is evidenced by the lowest mean number of transcripts per gene at 1.3 (others range from 1.7 to 2.3), and alternative splicing detected in only 18.1% of genes, significantly less than the 32.2% to 48.0% observed in the remaining species. Furthermore, P. nieuhofii has a substantially higher proportion of single-exon genes (12.5%), far exceeding the 3.5% to 4.4% found in the other catfish, which possess a more uniformly multi-exonic architecture. Structural measurements confirm this compactness: P. nieuhofii genes have a smaller average locus length (15,993.4 bp vs. 19,155.7 bp to 22,083.0 bp) and fewer distinct exons per gene (8.9 vs. 11.3 to 12.3). Its mean exon size is also the smallest at 180.2 bp (others range from 269.4 bp to 327.1 bp), resulting in a significantly shorter average transcript size (1,812.4 bp). 

 In terms of genome composition (Table 4), the P. nieuhofii genome has the lowest overall content dedicated to coding and genic regions. Exons constitute only 4% of the genome (48 Mb), which is notably low compared to the 8% to 13% seen elsewhere but exhibit the highest GC content at 51% (compared to 45%─46% in the others). Genes collectively occupy 44% of the genome (481 Mb), marking the smallest genic fraction (others range from 55% to 68%). Introns, totaling 233,110, account for 40% of the genome (434 Mb) with an average length of 1,862 bp, also representing the lowest proportional content in the comparison. The structural and compositional differences collectively indicate that the P. nieuhofii genome exhibits a more compact gene structure and less complexity in transcript diversity compared to the other catfish assemblies studied.”
